# Associations of spirometric *z*-scores with mobility, endurance-related performance, and functional lower-limb strength in community-dwelling older adults: a cross-sectional study

**DOI:** 10.3389/fpubh.2026.1913269

**Published:** 2026-09-23

**Authors:** Chuyuan Qiao, Yan Wang

**Affiliations:** School of Sports Medicine and Rehabilitation, Beijing Sport University, Beijing, China

**Keywords:** community-dwelling older adults, endurance-related performance, functional lower-limb strength, mobility, pulmonary function, *z*-score

## Abstract

**Background:**

Declining pulmonary function is closely associated with limitations in physical function among older adults. Previous studies have commonly used raw spirometric values or percent-predicted values, which are influenced by age, sex, height, and ethnicity, and have often focused on a single functional domain. Few studies have directly compared continuous standardized *z*-scores with lower limit of normal (LLN) classifications in relation to multidimensional physical performance, particularly among community-dwelling older adults in China. Within a public health framework for healthy aging, identifying community-assessable markers associated with early functional vulnerability may help inform risk stratification and preventive strategies to preserve mobility and independence. To examine the associations of spirometric *z*-scores with mobility, endurance-related performance, and functional lower-limb strength in community-dwelling older adults, and to assess the consistency between continuous *z*-score analyses and corresponding LLN-based classifications across physical performance outcomes.

**Methods:**

This cross-sectional study included 141 community-dwelling older adults in China aged ≥60 years. The primary exposures were *z*-scores for forced expiratory volume in 1 s (FEV₁), forced vital capacity (FVC), and FEV₁/FVC. Mobility was assessed using the Timed Up and Go (TUG) test and 4-m gait speed; endurance-related performance was assessed using the 2-min step test (2MST); overall physical function was assessed using the Short Physical Performance Battery (SPPB); and functional lower-limb strength was explored using the 30-s chair stand and the five-times sit-to-stand test. Spearman rank correlation and multivariable linear regression were used, with adjustment for age, sex, body mass index (BMI), smoking status, and number of comorbidities.

**Results:**

The mean age of the participants was 68.22 ± 5.75 years, and 80.9% were female. After adjustment for age, sex, BMI, smoking status, and number of comorbidities, each 1-unit increase in the FEV₁ and FVC *z*-scores was associated with a 0.162-s shorter TUG time (*β = −*0.162, 95% CI: −0.297 to −0.028) and a 0.167-s shorter TUG time (*β = −*0.167, 95% CI: −0.306 to −0.028), respectively. Each 1-unit increase in the FVC *z*-score was also associated with 3.605 more repetitions in the 2MST (*β =* 3.605, 95% CI: 1.297–5.913) and 1.143 more repetitions in the 30-s chair stand (*β =* 1.143, 95% CI: 0.568–1.718). The FEV₁/FVC *z*-score was not significantly associated with any core physical performance outcome. In the LLN-based analysis, participants with FVC below the LLN had a 0.461-s longer TUG time (*β =* 0.461, 95% CI: 0.061–0.861), completed 7.966 fewer repetitions in the 2MST (*β = −*7.966, 95% CI: −14.671 to −1.262), and completed 2.818 fewer repetitions in the 30-s chair stand (*β = −*2.818, 95% CI: −4.493 to −1.143) than those with FVC at or above the LLN. FEV₁/FVC below the LLN was not significantly associated with any core physical performance outcome. After outcome-specific false discovery rate correction, the associations of FEV₁ and FVC *z*-scores with TUG time (both *q* = 0.029) and of FVC *z*-score with 2MST performance (*q* = 0.006) remained statistically significant.

**Conclusion:**

Among community-dwelling older adults, spirometric *z*-scores—particularly the FVC *z*-score—were associated with mobility, endurance-related performance, and functional lower-limb strength. The consistent results obtained using continuous FVC *z*-scores and LLN-based classification suggest that FVC may provide complementary information when evaluating physical function. These findings should be confirmed in larger, multicenter longitudinal studies. From a public health perspective, standardized spirometry may complement brief physical-performance tests in community health assessments by helping to characterize older adults with concurrent respiratory and functional vulnerability. Longitudinal and intervention studies are needed to determine whether lower FVC predicts functional decline and whether respiratory muscle training, multicomponent exercise, or combined programs can preserve mobility and independence in later life.

## 1 Introduction

With the acceleration of population aging, declining lung function and its associated functional impacts have gradually become a significant issue in geriatric health management (1, 2). During the aging process, weakened lung elastic recoil, reduced thoracic compliance, diminished respiratory muscle function, and changes in the small airways can collectively lead to decreases in lung volume, expiratory flow rates, and ventilatory reserve (1, 2). In addition to diagnosed chronic respiratory diseases, community-dwelling older adults may also have subclinical lung function impairment or unrecognized spirometric abnormalities (3). Declining lung function in some community screening populations does not necessarily manifest as typical airflow obstruction; a non-obstructive low FVC pattern—characterized by a low FVC with a preserved FEV₁/FVC ratio—may also be relatively common (3, 4). Because early-stage lung function decline may be clinically subtle and its respiratory manifestations may be under-recognized, its potential impact on daily functional capacity and functional independence is easily underestimated in community screenings (1, 2, 4).

The association between reduced lung function and limitations in physical function may occur through multiple interrelated pathways. Lower forced expiratory volume in 1 s (FEV₁) and forced vital capacity (FVC) not only reflect reduced ventilatory capacity and lung volume reserves but may also be related to respiratory muscle function, thoracic mobility, physical activity levels, and overall health status (5, 6). During functional activities such as walking, turning, sit-to-stand transitions, and sustained lower-limb movement, insufficient ventilatory reserve may increase work of breathing, limit exercise tolerance, and reduce an individual’s ability to sustain activity (7). At the same time, age-related declines in muscle function, the burden of chronic diseases, and reduced physical activity can not only weaken lower-limb strength and mobility but may also affect respiratory muscle performance and spirometric measures (5, 6). Therefore, the association between pulmonary function and physical function may reflect not only local changes in the respiratory system but also the combined functional reserves of the respiratory, muscular, and neuromuscular systems (5, 6).

Physical performance is an important objective indicator of independent living ability and risk of functional decline in older adults (8, 9). Commonly used community-based tests include the Timed Up and Go (TUG) test and gait speed for mobility (8, 9), the 2-min step test (2MST) for endurance-related performance (10), the 30-s chair stand and five-times sit-to-stand tests for functional lower-limb strength (9, 11), and the Short Physical Performance Battery (SPPB) for overall physical function (9). Compared with a single test, multidimensional assessment can better identify different patterns of functional limitation and provides a basis for examining associations between lung function and specific domains of physical performance (9, 10).

Standardized methods are particularly crucial when interpreting pulmonary function results in older adults. The American Thoracic Society/European Respiratory Society (ATS/ERS) guidelines for pulmonary function testing and interpretation emphasize that results should be interpreted using appropriate reference equations, lower limits of normal, and *z*-scores, rather than relying solely on raw values, percent-predicted values, or fixed thresholds (12, 13). The Global Lung Function Initiative 2012 (GLI-2012) reference equations provide multiethnic spirometry reference values for individuals across the 3–95-year age range and allow for the calculation of corresponding *z*-scores and age-related lower limits of normal (14). Continuous spirometric *z*-scores account for differences in age, sex, height, and ethnicity while preserving information across the full distribution of pulmonary function; LLN classification identifies individuals whose values fall below the reference range (12, 14). These two measures may reflect the association between pulmonary function and physical function from different perspectives.

Previous studies have suggested a link between pulmonary function and physical function from various perspectives; however, the existing evidence still has three major limitations (3, 15–17). First, at the clinical level, the impact of subclinical pulmonary function decline and non-obstructive low FVC patterns in community-dwelling older adults on mobility, endurance-related performance, and functional lower-limb strength has not yet been fully understood. Anami et al. (3) found in a Japanese community-dwelling older adult population that preserved ratio impaired spirometry (PRISm) was associated with poorer physical performance; however, that study relied primarily on the PRISm classification and did not further analyze the association between continuous spirometric *z*-scores and various dimensions of physical function. Second, at the methodological level, previous studies have largely relied on raw values, percent-predicted values, or single categorical spirometric definitions, which are significantly influenced by differences in age, sex, height, and ethnicity (12, 14, 15, 17). The application of GLI-2012 standardized *z*-scores remains relatively limited, and there is a lack of systematic comparisons between continuous measures and corresponding LLN classification results. Kaneko et al. (15) investigated the relationships among respiratory function, physical performance, physical activity, and sedentary behavior in community-dwelling older adults, suggesting that lung volume and respiratory muscle function may be associated with physical performance; however, their focus was on respiratory muscle strength and physical activity behavior, and they did not use the GLI-2012 *z*-score framework to interpret pulmonary function. Finally, in terms of evidence, existing studies mostly focus on single functional dimensions such as muscle strength, gait speed, or sarcopenia. Petraglia et al. (16) recently analyzed the association between spirometric *z*-scores and muscle strength, providing a basis for the application of standardized pulmonary function measures in muscle function research; however, their functional outcomes were primarily concentrated on muscle strength and sarcopenia-related indicators, with insufficient coverage of multidimensional physical functions such as mobility, endurance-related performance, and functional lower-limb strength. In China, He et al. (17) reported associations of peak expiratory flow and percent-predicted peak expiratory flow with sarcopenia and its components, including gait speed, five-times sit-to-stand performance, and SPPB scores; however, they did not evaluate GLI-2012-based FEV₁, FVC, or FEV₁/FVC *z*-scores. Therefore, specific evidence regarding the associations of FEV₁, FVC, and FEV₁/FVC *z*-scores with multidimensional physical function in community-dwelling older adults in China remains limited.

Based on these knowledge gaps, the present study aimed to examine the associations of spirometric *z*-scores with mobility, endurance-related performance, and functional lower-limb strength in community-dwelling older adults, and to further determine whether the associations observed using LLN-based categorical definitions were consistent with those obtained using continuous *z*-scores. From a public health perspective, clarifying these associations may support the community-based identification of older adults with concurrent respiratory and functional vulnerability. Such evidence may also inform the design and targeting of preventive, non-pharmacological approaches aimed at preserving mobility and independence in later life. However, longitudinal and intervention studies are required to establish predictive utility and intervention effectiveness.

## 2 Methods

### 2.1 Study design and participants

This was a community-based cross-sectional study reported in accordance with the STROBE guidelines (18). It examined the associations of spirometric *z*-scores with mobility, endurance-related performance, and functional lower-limb strength in community-dwelling older adults. Participants were recruited by convenience sampling during health screenings and physical fitness assessments conducted in the Huayuanlu Community, Haidian District, Beijing, China, between November and December 2025. The present analysis used baseline data from these assessments. All participants completed standardized questionnaires, spirometry, and physical performance tests in the community setting. The study was approved by the Ethics Committee for Experimental Sports Science at Beijing Sport University (Approval No. 2025492H), and written informed consent was obtained from all participants.

An *a priori* sample size calculation was performed for the primary correlation analysis using a two-sided Fisher *z*-based test of a nonzero correlation (19). With an expected correlation coefficient of *r* = 0.250, a two-sided significance level of *α* = 0.05, and 80% statistical power, a minimum of 123 participants was required. This calculation served as a planning benchmark for the main correlation analysis and was not intended to provide confirmatory power for all secondary and exploratory analyses.

The inclusion criteria were as follows: (1) age ≥60 years; (2) provision of written informed consent; (3) long-term residence in the community; and (4) ability to understand the test instructions and independently complete the pulmonary function and physical performance assessments. The exclusion criteria were as follows: (1) inability to independently complete the pulmonary function or core physical performance assessments; (2) severe cognitive impairment, an acute disease episode, or another condition affecting testing safety; (3) a diagnosed severe chronic pulmonary disease, such as moderate-to-severe chronic obstructive pulmonary disease, interstitial lung disease, or pulmonary fibrosis; and (4) a condition preventing completion of the physical performance assessments, such as a lower-limb osteoarticular deformity or severe arthritis.

A total of 162 community-dwelling older adults underwent eligibility assessment. Following the on-site assessment, the final analytical sample was determined according to the eligibility criteria and data completeness. Participants were included in the descriptive analysis if they had valid pulmonary function data and data from at least one core physical performance assessment. Participants with missing key variables or obvious and unverifiable data-entry errors in the pulmonary function or physical performance data were excluded from the corresponding analyses. Ultimately, 141 participants were included in the descriptive analysis, exceeding the prespecified minimum sample size. The adjusted primary multivariable linear regression models used complete-case analysis and included 137 participants with complete data on spirometric *z*-scores, primary physical performance outcomes, age, sex, body mass index (BMI), smoking status, and number of comorbidities.

### 2.2 Pulmonary function assessment and *z*-score calculation

Pulmonary function testing was performed using an SP80B portable spirometer (CONTEC Medical Systems Co., Ltd., China). All tests were conducted by trained evaluators at the community site following a standardized protocol. Spirometry was performed with reference to the 2019 ATS/ERS technical standards (20). Participants were tested in a seated position while wearing a nose clip and using a disposable mouthpiece, with careful attention to preventing air leakage. Each participant performed at least three acceptable forced expiratory maneuvers. Acceptable maneuvers required a rapid and forceful start without hesitation, no coughing during the first second, no glottic closure or air leakage, and adequate completion of expiration according to the end-of-forced-expiration criteria. Repeatability was considered acceptable when the difference between the two largest FEV₁ values was ≤150 mL and the difference between the two largest FVC values was ≤150 mL. The largest FEV₁ and largest FVC obtained from acceptable maneuvers were used for analysis, irrespective of whether they occurred in the same maneuver (20). Maneuvers with substantial coughing, air leakage, delayed initiation, premature termination, incomplete expiration, glottic closure, or inadequate repeatability that did not improve after repeated attempts were considered to have failed the quality-control requirements and were excluded. All spirometric data included in the present analysis met the prespecified on-site quality-control criteria. Before testing each day, calibration verification was performed using a 3-L calibration syringe together with zero-flow calibration; ambient temperature and barometric-pressure corrections were applied automatically by the device. Operators received standardized training from respiratory specialists and the device manufacturer.

The recorded spirometric indices included FEV₁, FVC, FEV₁/FVC, and peak expiratory flow (PEF). Raw values of FEV₁, FVC, FEV₁/FVC, and PEF were used to describe participants’ spirometric characteristics. In the primary analyses, FEV₁, FVC, and FEV₁/FVC were entered separately as exposure variables in the form of *z*-scores to improve comparability across individuals differing in age, sex, height, and ethnicity (12, 14). FEV₁/FVC was expressed as a percentage for descriptive purposes, whereas its *z*-score was used in the analytical models to characterize airflow limitation relative to the reference distribution. Predicted values, *z*-scores, and lower limits of normal (LLNs) for FEV₁, FVC, and FEV₁/FVC were calculated using the Global Lung Function Initiative 2012 (GLI-2012) spirometry reference equations (14). Calculations were performed in R software (version 4.6.0; R Foundation for Statistical Computing, Vienna, Austria) using the rspiro package (version 0.5) (21). Based on participants’ age, sex, height, and ethnicity, the GLI-2012 North East Asian ethnic group was selected. The LLN was defined as the fifth percentile of the reference distribution, corresponding to a *z*-score of −1.645 (12, 14). The North East Asian equations were retained for the prespecified primary analysis. The South East Asian equations were used only in a reference-equation sensitivity analysis because they showed improved fit in a national Chinese sample (41). This sensitivity analysis assessed whether the spirometric distributions and exposure–outcome associations depended materially on the selected reference equation.

In the LLN-based analyses, low FEV₁, low FVC, and low FEV₁/FVC were defined as values below their corresponding LLNs (*z* < −1.645) and were examined separately as binary exposure variables. Spirometric patterns were also classified descriptively according to FVC and FEV₁/FVC relative to their LLNs. A normal spirometric pattern was defined as FEV₁, FVC, and FEV₁/FVC values at or above their respective LLNs. An FEV₁/FVC value below the LLN was classified as an obstructive spirometric pattern, regardless of FVC status. An FEV₁/FVC value at or above the LLN together with an FVC value below the LLN was classified as a non-obstructive low-FVC pattern. Isolated low FEV₁ was defined as an FEV₁ value below the LLN with both FVC and FEV₁/FVC at or above their respective LLNs. Because total lung capacity was not measured, an FVC value below the LLN was not interpreted as confirmation of restrictive ventilatory impairment (12).

### 2.3 Assessment of physical performance

Physical performance was assessed using standardized field tests and categorized according to functional attributes: mobility, endurance-related performance, overall physical function, and functional lower-limb strength. All tests were administered by trained evaluators following a standardized protocol; participants were briefed on the test procedures prior to testing and provided with demonstrations when necessary.

Mobility was assessed using the Timed Up and Go (TUG) test and a 4-m gait speed test. The TUG test was conducted according to the standard method proposed by Podsiadlo and Richardson, requiring participants to stand up from a chair, walk 3 meters at their usual pace, turn around, return to the chair, and sit down again (8). The test was performed twice, and the average completion time was recorded; a longer time indicates poorer mobility. The 4-m gait speed test was conducted in accordance with the SPPB and relevant standards for assessing physical function in older adults (9, 22). Participants were asked to walk 4 meters at their usual pace. The test was performed twice, and the shorter completion time was used to calculate gait speed; a higher value indicates better walking ability.

Endurance-related performance was assessed using the 2-min step test (2MST), which was conducted in accordance with the Senior Fitness Test system proposed by Rikli and Jones (10). The target knee-lift height was defined as the midpoint of the line connecting the iliac crest and the midpoint of the patella. Participants performed alternating steps for 2 min; a valid step was counted when the right knee reached or exceeded the target height, and a higher number of completed steps indicated better endurance-related performance.

Overall physical function was assessed using the Short Physical Performance Battery (SPPB). The SPPB, conducted according to the standard method proposed by Guralnik et al. (9), consists of three components: balance, gait speed, and sit-to-stand ability; a higher total score indicates better overall lower-limb function. Functional lower-limb muscle strength was assessed in exploratory analyses using the 30-s chair stand and the five-times sit-to-stand test. The tests were conducted using a chair without armrests with a seat height of approximately 43 cm; participants placed both feet flat on the floor and crossed their arms over their chest. The 30-s chair stand was performed according to the method described by Jones (11), and the number of complete sit-to-stand repetitions performed during a single 30-s trial was recorded. The five-times sit-to-stand test was conducted following the chair-rise procedure of the SPPB, and the time required to complete five consecutive sit-to-stand transitions was recorded when the participant reached a fully upright position after the fifth repetition (9). A higher number of repetitions in the former test and a shorter completion time in the latter indicate better functional lower-limb strength.

These field tests are widely used for the assessment of physical performance in older adults. Evidence supports the reliability of the TUG, SPPB, and 4-m gait speed test in community-dwelling older populations, although the strength of evidence for some other measurement properties varies across tests (22). The SPPB and five-times sit-to-stand test have also been extensively applied in large samples of community-dwelling older adults in China (23, 24).

### 2.4 Covariates

Based on previous evidence and physiological considerations, age, sex, and body mass index (BMI) were selected as key demographic and anthropometric covariates because they are associated with both pulmonary function and physical performance in older adults (25). Although age and sex are incorporated into the GLI-2012 *z*-score calculation to standardize the spirometric exposure, they were retained as covariates because this standardization does not account for their independent associations with physical-performance outcomes. Smoking status was additionally considered because cigarette smoking is an established determinant of accelerated pulmonary function decline (26). The number of comorbidities was used to represent overall chronic disease burden, which may adversely affect mobility, muscle strength, and other dimensions of physical performance among older adults (27). The adjusted model therefore included age, sex, BMI, smoking status, and number of comorbidities.

### 2.5 Statistical analysis

All statistical analyses were performed using R software (version 4.6.0; R Foundation for Statistical Computing, Vienna, Austria). Continuous variables were presented as means and standard deviations, and categorical variables were presented as frequencies and percentages. Complete-case analysis was used. The descriptive analysis and adjusted regression models included 141 and 137 participants, respectively. Given the minimal amount of missing data, no data imputation was performed. Each supplementary model was based on participants with complete data for the variables included in that specific analysis; therefore, sample sizes varied slightly across models.

Given the skewed distributions, potential outliers, and tied ranks observed in some physical performance outcomes, Spearman rank correlation analysis was used to assess the bivariate associations of FEV₁, FVC, and FEV₁/FVC *z*-scores with physical performance outcomes (28). Multiple linear regression was subsequently used to examine the adjusted associations between spirometric *z*-scores and physical performance. The adjusted model included age, sex, BMI, smoking status, and number of comorbidities. Each spirometric *z*-score was entered into a separate regression model. Scatter plots and residual-versus-fitted plots were inspected to assess linearity and homoscedasticity, and residual Q–Q plots were examined to evaluate the normality of residuals. Outliers and influential observations were also assessed, and no substantial violations of the regression assumptions were identified. As a sensitivity analysis, the adjusted primary models were additionally adjusted for self-reported physical activity, categorized as no regular exercise, occasional exercise, or regular exercise.

The core outcomes were TUG time, 4-m gait speed, and 2MST performance. SPPB score was treated as a secondary outcome, whereas the 30-s chair stand and five-times sit-to-stand tests were treated as exploratory outcomes. This hierarchy was specified *a priori* to reflect the study’s primary focus on domain-specific measures of mobility and endurance-related performance. Although the SPPB is a well-validated multidomain measure, it was designated as a secondary outcome because its composite score incorporates balance, gait speed, and chair-rise performance and therefore partially overlaps with the separately assessed 4-m gait speed and sit-to-stand tests. Supplementary analyses examined the associations of FEV₁, FVC, and FEV₁/FVC below the LLN with physical performance, using participants with the corresponding spirometric index at or above the LLN as the reference group. Spirometric patterns were summarized using frequencies and percentages. To facilitate comparison of effect sizes across physical performance outcomes, the regression coefficients and corresponding 95% CIs for the continuous FVC *z*-score and FVC-below-LLN models were divided by the standard deviation of the corresponding outcome. TUG time and five-times sit-to-stand time were reverse-coded so that positive standardized coefficients consistently indicated better physical performance. The key continuous and LLN-based analyses were repeated using the South East Asian equations. The prespecified primary exposures were the FEV₁, FVC, and FEV₁/FVC *z*-scores, and the prespecified primary outcomes were TUG time, 4-m gait speed, and 2MST performance. Because the three outcomes represented distinct physical-performance domains, the three associations involving the spirometric *z*-scores were treated as a separate analytical family for each primary outcome. The Benjamini–Hochberg procedure was applied within each outcome-specific family to control the false discovery rate, with *q* < 0.05 considered statistically significant. SPPB, chair-stand, LLN-based, and spirometric-pattern analyses were considered secondary or exploratory and were not included in this correction.

For secondary and exploratory analyses outside the prespecified primary FDR families, raw *p*-values were reported without multiplicity correction and interpreted cautiously (29). Accordingly, findings were interpreted cautiously by considering the direction and magnitude of the associations, 95% *CI*s, and consistency across related analyses, rather than relying solely on whether the *p*-value crossed a fixed threshold (30). All statistical tests were two-sided. For the prespecified primary associations, statistical significance was determined using FDR-adjusted *q* < 0.05.

## 3 Results

### 3.1 Characteristics of study participants

This study evaluated a total of 162 community-dwelling older adults. Of these, 21 were excluded because they did not meet the on-site inclusion criteria, their pulmonary function test results did not meet quality control requirements, they were unable to complete the primary physical performance tests, or key variables were missing. Ultimately, 141 Chinese community-dwelling older adults aged ≥60 years were included in the descriptive analysis. The adjusted primary regression model included 137 participants with complete outcome, exposure, and covariate data. The participant inclusion flowchart is shown in Figure 1.

**Figure 1:**
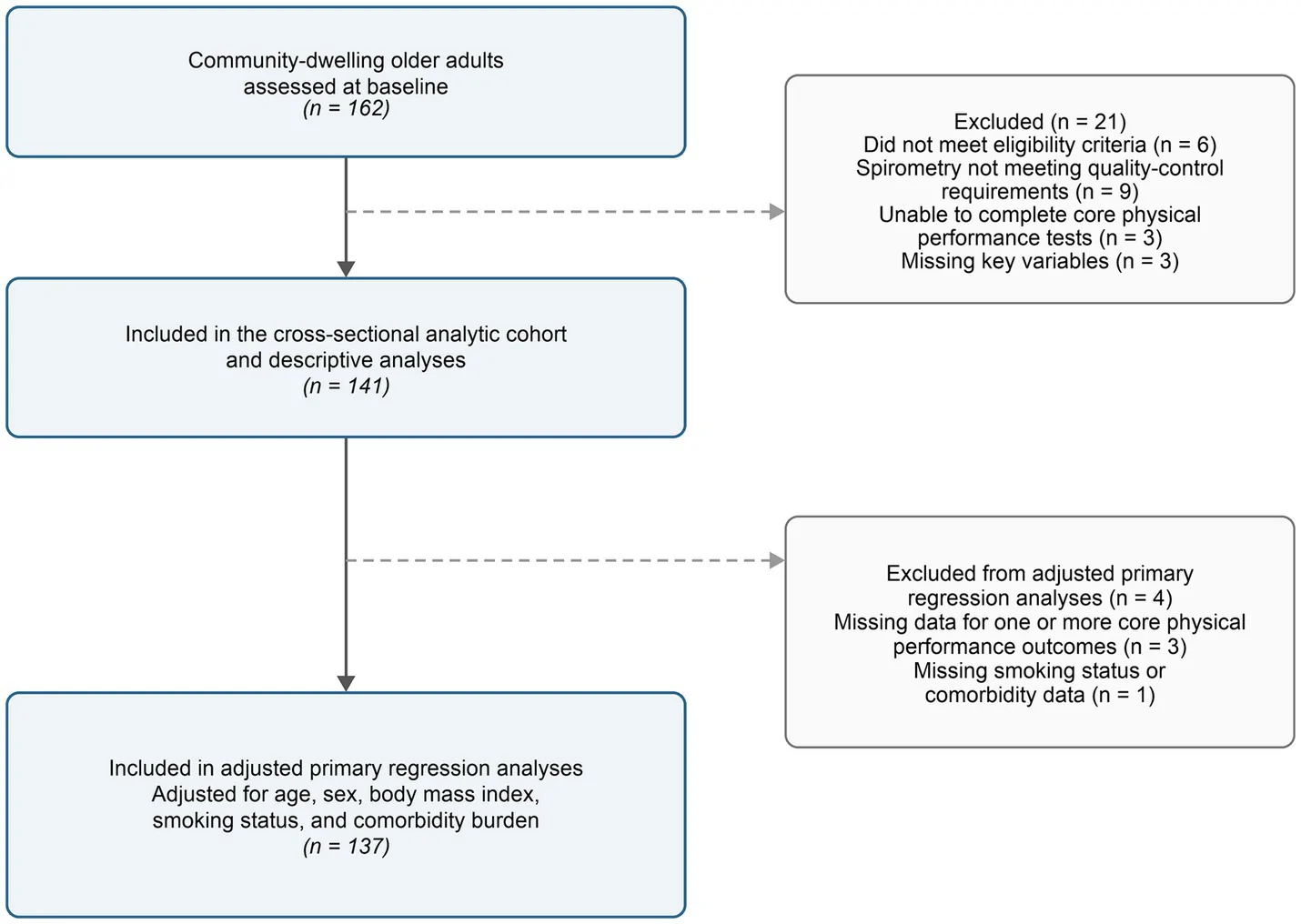
Flow diagram of participant inclusion.

The 141 older adults included in the descriptive analysis had a mean age of 68.22 ± 5.75 years, of whom 114 (80.9%) were women. The mean height of the participants was 161.77 ± 7.03 cm, the mean weight was 63.61 ± 11.23 kg, and the mean body mass index was 24.20 ± 3.41 kg/m^2^. Most participants were never smokers (94.2%), and 22.5% consumed alcohol; 35.3% exercised regularly, while 44.6% exercised occasionally. Participants with two or more chronic conditions accounted for 74.8%, and those taking 1–4 medications accounted for 69.6%. Regarding physical function, the participants’ mean Timed Up and Go (TUG) was 6.90 ± 1.22 s, their 4-m gait speed was 1.32 ± 0.22 m/s, the number of repetitions completed in the 2MST was 102.79 ± 19.51, the number of repetitions completed in the 30-s chair stand was 18.94 ± 5.11, and the time for the five-times sit-to-stand test was 9.38 ± 3.42 s. Detailed characteristics of the study participants are shown in Table 1.

**Table 1:** Characteristics of the study participants.

Characteristic	Overall (*N* = 141)
Age, years	68.22 ± 5.75
Sex	
Male	27 (19.1%)
Female	114 (80.9%)
Height, cm	161.77 ± 7.03
Weight, kg	63.61 ± 11.23
Body mass index, kg/m^2^	24.20 ± 3.41
Smoking status	
Never	131 (94.2%)
Former	7 (5.0%)
Current	1 (0.7%)
Alcohol consumption	31 (22.5%)
Exercise frequency	
None	28 (20.1%)
Occasional	62 (44.6%)
Regular	49 (35.3%)
Number of medications	
0	35 (25.9%)
1–4	94 (69.6%)
≥5	6 (4.4%)
Number of comorbidities	
0	11 (7.9%)
1	24 (17.3%)
≥2	104 (74.8%)
FVC, L	2.16 ± 0.57
FEV₁, L	1.80 ± 0.56
FEV₁/FVC, %	83.06 ± 12.12
PEF, L/s	3.68 ± 2.11
FEV₁ *z*-score	−1.43 ± 1.42
FVC *z*-score	−2.09 ± 1.39
FEV₁/FVC *z*-score	0.85 ± 2.05
FEV₁ below the lower limit of normal (LLN)	64 (45.4%)
FVC below the lower limit of normal (LLN)	91 (64.5%)
FEV₁/FVC below the lower limit of normal (LLN)	17 (12.1%)
Spirometric pattern	
Normal spirometric pattern	40 (28.4%)
Obstructive spirometric pattern	17 (12.1%)
Non-obstructive low-FVC pattern	78 (55.3%)
Isolated low FEV₁	6 (4.3%)
TUG time, s	6.90 ± 1.22
4-m gait speed, m/s	1.32 ± 0.22
2-min step test, repetitions	102.79 ± 19.51
30-s chair stand, repetitions	18.94 ± 5.11
Five-times sit-to-stand, s	9.38 ± 3.42
SPPB score	
8	2 (1.4%)
9	2 (1.4%)
10	6 (4.3%)
11	15 (10.8%)
12	114 (82.0%)

Data are presented as mean ± SD or n (%). Percentages were calculated using non-missing data; denominators therefore vary across variables. LLN, lower limit of normal; FVC, forced vital capacity; FEV₁, forced expiratory volume in 1 s; PEF, peak expiratory flow; TUG, Timed Up and Go; SPPB, Short Physical Performance Battery.

### 3.2 Pulmonary function characteristics and distribution of LLN-based spirometry patterns

The mean FVC, FEV₁, and FEV₁/FVC of the study participants were 2.16 ± 0.57 L, 1.80 ± 0.56 L, and 83.06% ± 12.12%, respectively; the mean PEF was 3.68 ± 2.11 L/s. The *z*-scores for FEV₁, FVC, and FEV₁/FVC, calculated using the GLI-2012 reference equations, were −1.43 ± 1.42, −2.09 ± 1.39, and 0.85 ± 2.05, respectively.

Using a *z*-score < −1.645 as the criterion for lower limit of normal (LLN), 64 participants (45.4%), 91 (64.5%), and 17 (12.1%) had FEV₁, FVC, and FEV₁/FVC below the LLN, respectively. After mutually exclusive classification based on the results of FEV₁, FVC, and FEV₁/FVC relative to the LLN, 40 subjects (28.4%) exhibited a normal spirometric pattern, 17 (12.1%) exhibited an obstructive spirometric pattern, and 78 (55.3%) exhibited a non-obstructive low-FVC pattern, while 6 (4.3%) exhibited isolated low FEV₁. Overall, low spirometric indices in this sample were primarily driven by FVC below the LLN and the non-obstructive low FVC pattern, whereas the proportion of obstructive patterns indicated by FEV₁/FVC below the LLN was relatively low; see Supplementary Figure 1 for the distributions of the *z*-scores and the proportions below the LLN. Detailed characteristics of participants’ pulmonary function are shown in Table 1. In the reference-equation sensitivity analysis, use of the GLI-2012 South East Asian equations shifted the mean FVC *z*-score from −2.09 to −1.01 and reduced the proportion with FVC below the LLN from 64.5% (91/141) to 26.2% (37/141); the proportion with a non-obstructive low-FVC pattern decreased from 55.3% (78/141) to 23.4% (33/141) (Supplementary Table 4).

### 3.3 Bivariate correlation analysis

Spearman rank correlation analysis showed that the FEV₁ *z*-score was negatively correlated with TUG time (*ρ = −*0.333, *p <* 0.001) and positively correlated with 4-m gait speed (*ρ =* 0.176, *p =* 0.039); correlations with the 2MST and SPPB were not statistically significant.

The FVC *z*-score was negatively correlated with TUG time (*ρ = −*0.234, *p =* 0.006) and positively correlated with 4-m gait speed (*ρ =* 0.196, *p =* 0.021) and 2MST (*ρ =* 0.202, *p =* 0.018). The FEV₁/FVC *z*-score was weakly negatively correlated only with TUG time (*ρ = −*0.200, *p =* 0.019). The results of the correlation analysis between spirometric *z*-scores and key indicators of physical function are shown in Table 2, and representative bivariate scatter plots are shown in Figures 2A–C. These unadjusted correlations were descriptive; their raw *p* values were not used for confirmatory inference.

**Table 2:** Spearman correlations between spirometric *z*-scores and selected physical performance outcomes.

**Spirometric *z*-score**	**TUG time**	**4-m gait speed**	**2MST**	**SPPB**
FEV₁ *z*-score	−0.333 (<0.001)	0.176 (0.039)	0.147 (0.085)	0.041 (0.631)
FVC *z*-score	−0.234 (0.006)	0.196 (0.021)	0.202 (0.018)	0.082 (0.339)
FEV₁/FVC *z*-score	−0.200 (0.019)	0.017 (0.847)	−0.036 (0.678)	−0.043 (0.618)

Values are Spearman correlation coefficients (ρ) with raw *p*-values in parentheses. These unadjusted analyses are descriptive and were not included in the prespecified FDR correction. TUG, Timed Up and Go; 2MST, 2-min step test; SPPB, Short Physical Performance Battery; FEV₁, forced expiratory volume in 1 s; FVC, forced vital capacity.

**Figure 2:**
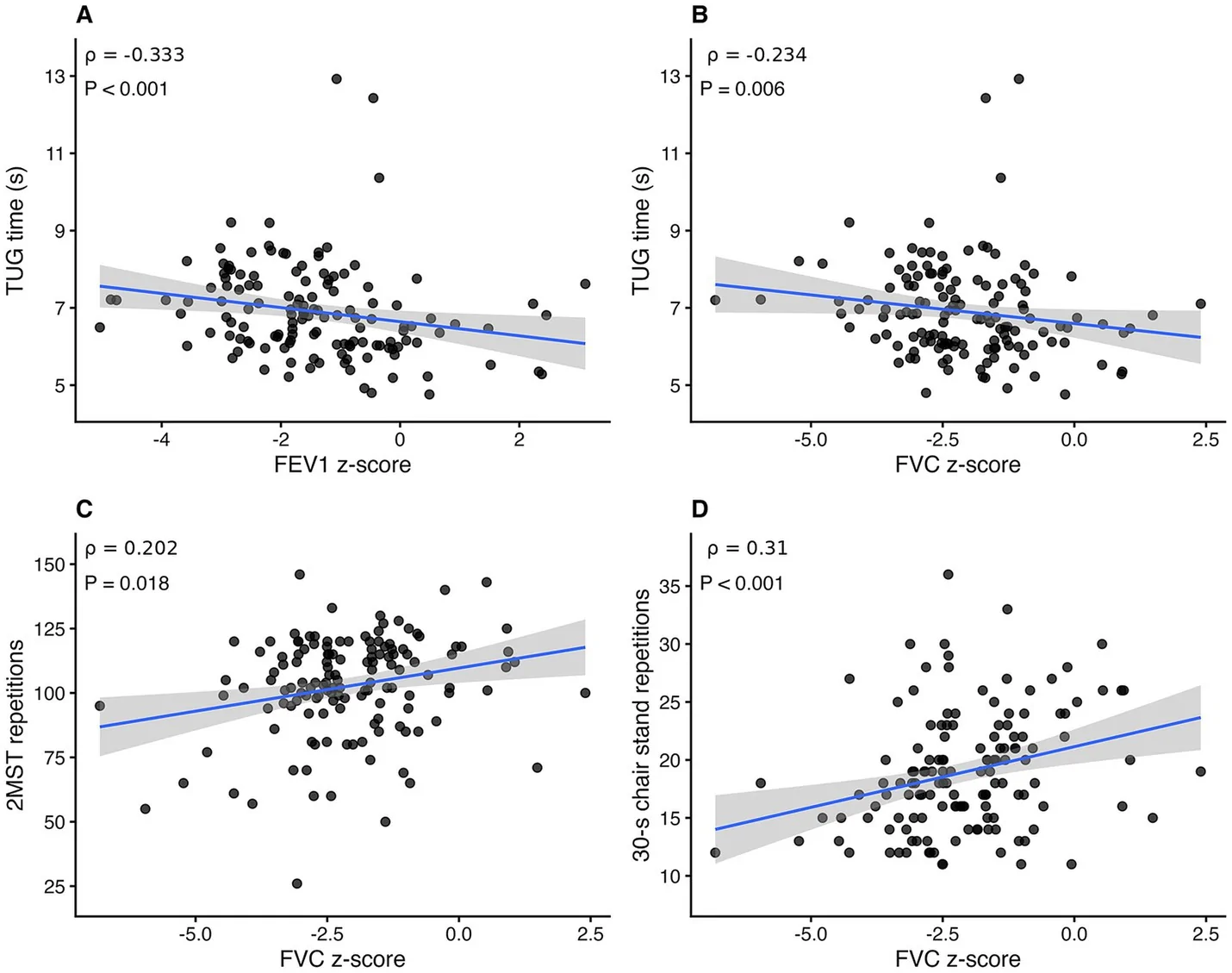
Associations of spirometric *z*-scores with mobility, endurance-related performance, and functional lower-limb strength outcomes. Scatter plots show the associations between **(A)** FEV₁ *z*-score and TUG time, **(B)** FVC *z*-score and TUG time, **(C)** FVC *z*-score and 2MST repetitions, and **(D)** FVC *z*-score and 30-s chair stand repetitions. Blue lines represent fitted linear trends, and shaded areas represent 95% confidence intervals. Spearman’s correlation coefficients (ρ) and the corresponding raw p-values are displayed in each panel. TUG, Timed Up and Go; 2MST, 2-min step test; FEV₁, forced expiratory volume in 1 s; FVC, forced vital capacity. The displayed p-values are raw and descriptive; confirmatory inference is based on the adjusted FDR-controlled analyses.

### 3.4 Multiple linear regression analysis

In the adjusted model, higher FEV₁ and FVC *z*-scores were associated with shorter TUG time (FEV₁: *β* = −0.162, 95% CI: −0.297 to −0.028, raw *p* = 0.019, *q* = 0.029; FVC: *β* = −0.167, 95% CI: −0.306 to −0.028, raw *p* = 0.019, *q* = 0.029). A higher FVC *z*-score was also associated with better 2MST performance (*β* = 3.605, 95% CI: 1.297–5.913, raw *p* = 0.002, *q* = 0.006). These three associations met the prespecified FDR-adjusted significance threshold of *q* < 0.05. The FEV₁/FVC *z*-score did not meet this threshold for any primary outcome. Results are presented in Table 3, Figures 3A–C, and Supplementary Table 5. Additional adjustment for physical activity modestly attenuated the associations but yielded similar directions and magnitudes: FEV₁ *z*-score with TUG time (*β* = −0.145, 95% CI: −0.277 to −0.013, raw *p* = 0.031, *q* = 0.051), FVC *z*-score with TUG time (*β* = −0.148, 95% CI: −0.285 to −0.011, raw *p* = 0.034, *q* = 0.051), and FVC *z*-score with 2MST performance (*β* = 3.065, 95% CI: 0.863–5.267, raw *p* = 0.007, *q* = 0.020). Thus, after outcome-specific FDR correction, the FVC–2MST association remained significant, whereas both TUG associations were borderline (Supplementary Table 6).

**Table 3:** Multivariable linear regression models for associations between spirometric *z*-scores and physical performance outcomes.

Outcome	Exposure	Adjusted *β* (95% CI)	Raw *p*-value	FDR-adjusted *q*	*n*
Timed Up and Go, s	FEV₁ *z*-score	−0.162 (−0.297, −0.028)	0.019	0.029	137
Timed Up and Go, s	FVC *z*-score	−0.167 (−0.306, −0.028)	0.019	0.029	137
Timed Up and Go, s	FEV₁/FVC *z*-score	−0.027 (−0.123, 0.069)	0.575	0.575	137
4-m gait speed, m/s	FEV₁ *z*-score	0.015 (−0.011, 0.041)	0.247	0.371	137
4-m gait speed, m/s	FVC *z*-score	0.025 (−0.002, 0.052)	0.064	0.192	137
4-m gait speed, m/s	FEV₁/FVC *z*-score	−0.004 (−0.023, 0.014)	0.652	0.652	137
2-min step test, repetitions	FEV₁ *z*-score	2.277 (−0.004, 4.558)	0.050	0.075	137
2-min step test, repetitions	FVC *z*-score	3.605 (1.297, 5.913)	0.002	0.006	137
2-min step test, repetitions	FEV₁/FVC *z*-score	−0.440 (−2.058, 1.178)	0.591	0.591	137

FEV₁, forced expiratory volume in 1 s; FVC, forced vital capacity; CI, confidence interval; FDR, false discovery rate. The adjusted model included age, sex, BMI, smoking status, and comorbidity burden. Each spirometric *z*-score was entered into a separate model. The Benjamini–Hochberg procedure was applied separately within each primary-outcome family comprising the three spirometric *z*-score associations; *q* < 0.05 was considered statistically significant.

**Figure 3:**
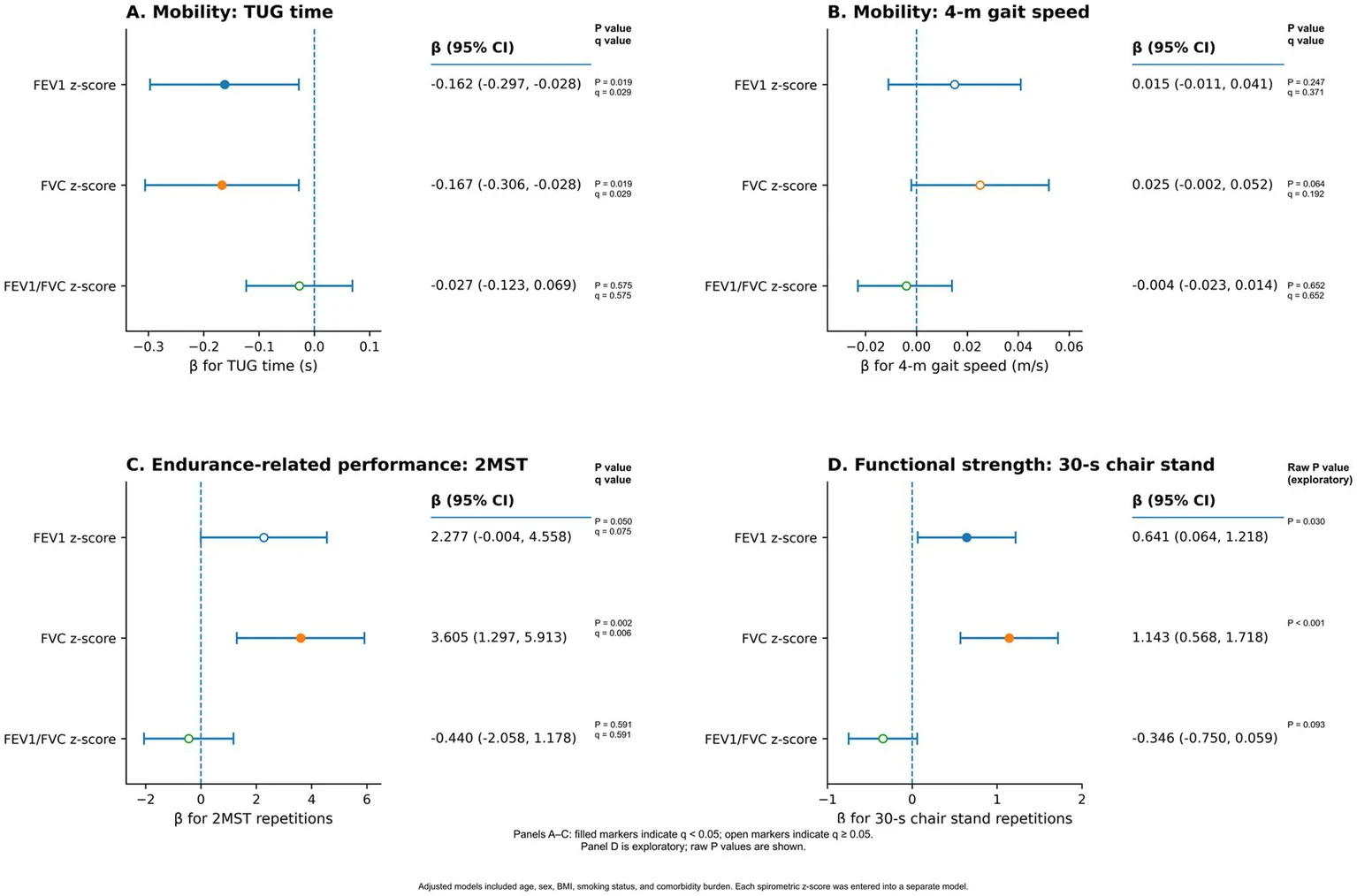
Adjusted associations between spirometric *z*-scores and mobility, endurance-related performance, and functional lower-limb strength outcomes. Forest plots show adjusted regression coefficients for TUG time **(A)**, 4-m gait speed **(B)**, 2MST repetitions **(C)**, and 30-s chair stand repetitions **(D)**. Models were adjusted for age, sex, BMI, smoking status, and number of comorbidities; each spirometric *z*-score was entered into a separate model. Points indicate regression coefficients, and horizontal lines indicate 95% confidence intervals. Panels **(A–C)** show raw *p-*values and outcome-specific FDR-adjusted q values; filled circles indicate *q* < 0.05 and open circles indicate *q* ≥ 0.05. Panel **(D)** is exploratory and shows raw *p* values only. TUG, Timed Up and Go; 2MST, 2-min step test; FEV₁, forced expiratory volume in 1 s; FVC, forced vital capacity; BMI, body mass index; FDR, false discovery rate.

### 3.5 Secondary and exploratory analyses

Multivariable linear regression analysis using the SPPB as a secondary outcome showed that, after adjusting for age, sex, BMI, smoking status, and number of comorbidities, none of the FEV₁, FVC, or FEV₁/FVC *z*-scores were significantly associated with SPPB scores (Supplementary Table 1). Given that SPPB scores in this sample were highly concentrated at the maximum score, this result may be influenced by a ceiling effect.

In an exploratory analysis of functional lower-limb strength, the adjusted model showed that both FEV₁ and FVC *z*-scores were associated with more 30-s chair stand repetitions (FEV₁: *β =* 0.641, 95% CI: 0.064–1.218, *p =* 0.030; *FVC*: *β =* 1.143, 95% CI: 0.568–1.718, *p <* 0.001), whereas the association of the FEV₁/FVC *z*-score with 30-s chair stand repetitions did not reach statistical significance (*β = −*0.346, 95% CI: −0.750–0.059, *p =* 0.093). *Neither* FEV₁, FVC, nor the FEV₁/FVC *z*-score was significantly associated with five-times sit-to-stand time; among these, the FVC *z*-score exhibited a negative trend but did not reach statistical significance (*β = −*0.350, 95% CI: −0.755 to 0.055, *p =* 0.090). Results of the exploratory analyses of functional lower-limb strength are presented in Supplementary Table 2 and Figure 3D, while the corresponding scatter plot for the association between FVC *z*-score and 30-s chair stand repetitions is shown in Figure 2D. The *p*-values in these exploratory analyses are raw and were not multiplicity-corrected.

### 3.6 Associations of spirometric indices below the lower limit of normal with physical performance

In the LLN-based classification analysis, after further adjusting for age, sex, BMI, smoking status, and number of comorbidities, an FVC below the LLN was associated with a longer TUG time (*β =* 0.461, 95% CI: 0.061–0.861, *p =* 0.024), fewer 2MST repetitions (*β = −*7.966, 95*%* CI: −14.671 to −1.262, *p =* 0.020), and a lower number of 30-s chair stand repetitions (*β = −*2.818, 95% CI: −4.493 to −1.143, *p =* 0.001). An FVC below the LLN was associated with slower 4-m gait speed but did not reach statistical significance (*p =* 0.065); it was not significantly associated with SPPB scores or the time taken for the five-times sit-to-stand test. An FEV₁ below the LLN was not significantly associated with TUG time in the adjusted analysis. An FEV₁/FVC ratio below the LLN was not significantly associated with any physical performance outcomes. Overall, FVC below the LLN showed the most consistent associations with physical performance and generally aligned with the findings from the continuous FVC *z*-score analysis (see Supplementary Table 3). Figure 4 shows the adjusted, outcome-standardized effects of continuous FVC *z*-scores and FVC below the LLN on various physical performance outcomes. Using the South East Asian equations, the continuous FVC *z*-score remained associated with TUG time (*β* = −0.201, 95% CI: −0.372 to −0.031, *p* = 0.021) and 2MST performance (*β* = 4.274, 95% CI: 1.442–7.106, *p* = 0.003). South East Asian equation-defined low FVC remained associated with fewer 2MST repetitions (*β* = −9.638, 95% CI: −16.884 to −2.391, *p* = 0.010), whereas its associations with TUG time and gait speed were not statistically significant (Supplementary Table 4). The *p*-values reported for these secondary and sensitivity analyses are raw and were interpreted together with effect estimates and 95% CIs.

**Figure 4:**
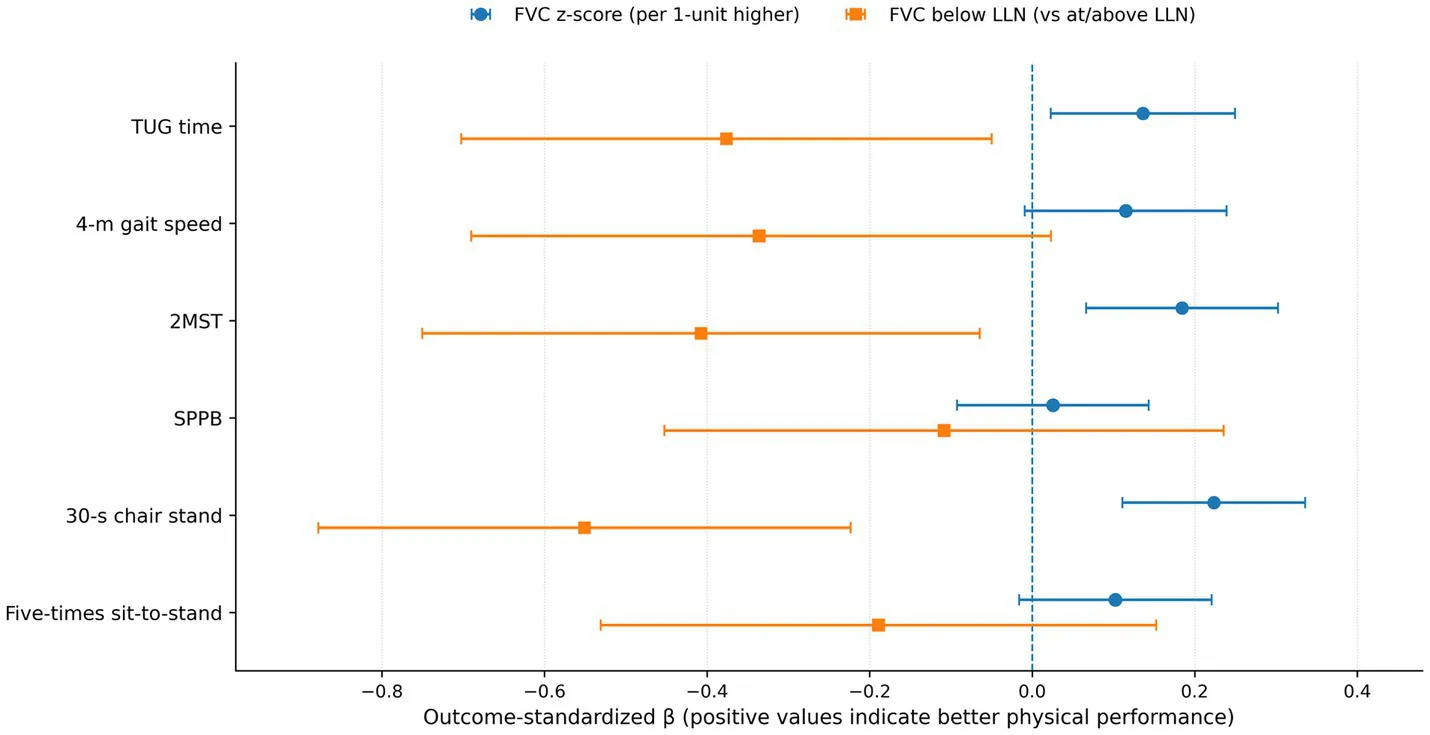
Outcome-standardized associations of continuous FVC *z*-score and LLN-defined low FVC with physical performance outcomes. Adjusted, outcome-standardized regression coefficients and 95% confidence intervals are shown. Models were adjusted for age, sex, body mass index, smoking status, and comorbidity burden. TUG time and five-times sit-to-stand time were reverse-coded so that positive coefficients consistently indicate better physical performance. Marker fill does not encode statistical significance; interpretation emphasizes effect estimates and 95% confidence intervals. LLN, lower limit of normal; FVC, forced vital capacity; TUG, Timed Up and Go; 2MST, 2-min step test; SPPB, Short Physical Performance Battery.

## 4 Discussion

### 4.1 Key findings

Among the prespecified primary analyses, higher FEV₁ and FVC *z*-scores were associated with shorter TUG times, and a higher FVC *z*-score was associated with better 2MST performance after outcome-specific FDR correction. None of the spirometric *z*-scores was associated with 4-m gait speed after FDR correction, and the FEV₁/FVC *z*-score was not associated with any primary outcome. Overall, FVC showed the most consistent associations with the primary mobility and endurance-related outcomes.

### 4.2 Comparison with previous studies and study contributions

The findings of the present study are broadly consistent with previous evidence linking pulmonary function to physical performance, while extending this evidence through the use of standardized spirometric indices and a broader range of functional outcomes. In a national sample of aging adults from Korea, Choi et al. (31) reported that abnormal SPPB performance was associated with abnormal pulmonary function and that gait speed was significantly or marginally associated with FEV₁ and FVC among participants aged 60 years or older. Similarly, Simpson et al. (32) found that lower FEV₁ and FVC were associated with slower 4-m walking performance and greater self-reported physical disability among community-dwelling older women. Kaneko et al. (15) also reported associations of respiratory function, including lung volumes and respiratory muscle strength, with physical performance in ambulatory community-dwelling older adults. Evidence from older patients with COPD further showed that greater pulmonary function impairment was associated with poorer SPPB performance, shorter 6-min walk distance, and greater mobility limitations, although this disease-specific evidence may not be directly generalizable to community populations without diagnosed respiratory disease (33). In terms of magnitude, a one-unit higher FVC *z*-score corresponded to a 0.167-s shorter TUG time and 3.605 additional 2MST repetitions in the primary models. Using the adjusted regression coefficient for TUG time (*β* = −0.167 s per one-unit higher FVC *z*-score), a clinically important TUG difference of 0.8–1.4 s would algebraically correspond to an approximately 4.8–8.4-unit higher FVC *z*-score (0.8/0.167 to 1.4/0.167). Thus, the TUG difference associated with a one-unit higher FVC *z*-score was substantially smaller than this published clinically important range. However, the 0.8–1.4-s threshold was derived from patients with hip or knee osteoarthritis (34), and no well-established TUG MCID is currently available for high-functioning community-dwelling older adults; a recent systematic review rated the evidence for TUG responsiveness in community-dwelling older adults as very low (22). Moreover, because this study was cross-sectional, the calculated 4.8–8.4-unit value represents a between-participant contrast under the fitted linear model rather than the within-person improvement in FVC required to produce a clinically meaningful change in TUG. This *post hoc* algebraic calculation should therefore be interpreted as a contextual assessment of effect magnitude rather than a treatment threshold.

Evidence based on standardized spirometric measures remains more limited. In a large multicenter Canadian cohort, Duong et al. observed graded associations between lower GLI-based FEV₁ categories and poorer gait speed, TUG performance, standing balance, and handgrip strength, with broadly consistent findings across age, sex, smoking, and spirometric subgroups (35). More recently, Petraglia et al. (16) found that FEV₁ and FVC *z*-scores were positively associated with handgrip strength after adjustment for demographic characteristics, physical activity, and comorbidities, and that lower spirometric indices were observed among participants classified as having probable sarcopenia according to grip-strength thresholds. The present study extends these findings by simultaneously evaluating TUG, 2MST, gait speed, SPPB, and chair stand performance and by directly comparing continuous FEV₁, FVC, and FEV₁/FVC *z*-scores. In particular, the association of the FVC *z*-score with mobility, endurance-related performance, and functional lower-limb strength provides additional evidence from a community-dwelling older population in East Asia.

Evidence from Japan has also suggested that preserved ratio impaired spirometry (PRISm) may be accompanied by selected deficits in physical performance among community-dwelling older adults. Anami et al. (3) observed poorer core muscle endurance among participants with PRISm than among those with normal spirometry, although differences in several other physical performance measures were not statistically significant. Because that study primarily compared participants according to a dichotomous spirometric classification, it did not characterize associations across the continuous distribution of pulmonary function. In the present study, FVC below the LLN was associated with poorer TUG, 2MST, and 30-s chair stand performance, while the continuous FVC *z*-score showed directionally consistent associations across these outcomes. These findings suggest that the relationship between FVC and physical performance may be observable across a continuum rather than only after FVC falls below the LLN. Continuous *z*-scores quantify an individual’s position within the reference distribution and retain information that may be lost through dichotomization, whereas LLN-based classification provides a clinically interpretable threshold for identifying values outside the reference range (12, 14). The two approaches should therefore be regarded as complementary rather than interchangeable.

Evidence from China has generally relied on PEF, absolute spirometric values, percent-predicted values, or categorical spirometric patterns and has often focused on individual outcomes or sarcopenia-related constructs. Using data from the China Health and Retirement Longitudinal Study, He et al. (17) examined the associations of PEF and percent-predicted PEF with sarcopenia and its components, including handgrip strength, gait speed, five-times chair stand performance, and SPPB score. In addition, a recent study of 2,526 community-dwelling adults in Beijing found that handgrip strength, gait speed, and skeletal muscle mass were positively associated with FEV₁ and FVC, while a longer five-times sit-to-stand time was associated with poorer spirometric indices and higher odds of abnormal spirometric patterns (36). However, these Chinese studies did not evaluate continuous GLI-based FEV₁, FVC, and FEV₁/FVC *z*-scores in relation to mobility, endurance-related performance, and functional lower-limb strength within the same analytical framework. By applying GLI-2012 standardized *z*-scores, which account for age, sex, height, and population group, and by simultaneously assessing multiple physical performance domains, the present study improves comparability across individuals and provides additional evidence regarding the association between FVC and multidimensional physical performance among community-dwelling older adults in China (14).

### 4.3 Possible mechanisms underlying the association between FVC and physical function

Pulmonary function and physical function may share pathways related to age-associated skeletal muscle decline. The diaphragm and intercostal muscles are skeletal muscles and may be affected by aging, physical inactivity, undernutrition, inflammation, and other processes that also contribute to peripheral sarcopenia. Age-related sarcopenic processes may affect respiratory and peripheral skeletal muscles through partly shared pathways, including physical inactivity, undernutrition, inflammation, and impaired anabolic signaling (37). Supporting this concept, Ohara et al. (38) found that community-dwelling older adults with sarcopenia had lower maximal inspiratory and expiratory pressures than those without sarcopenia, and that respiratory muscle strength was associated with handgrip strength, gait speed, and muscle mass. FVC is determined not only by lung volume and thoracic mechanics but also by the ability to inspire fully, generate an effective forced expiration, and sustain adequate effort throughout the maneuver (20). A lower FVC may therefore partly reflect reductions in respiratory muscle function or broader physiological reserve that occur in parallel with peripheral muscle decline. Such shared processes could contribute to the observed associations of FVC with mobility, endurance-related performance, and repeated sit-to-stand ability, although respiratory muscle strength was not directly measured in the present study.

A lower FVC may indicate reduced capacity to accommodate the increased ventilatory requirements of sustained physical activity. Normal aging is accompanied by reduced lung elastic recoil, decreased chest-wall compliance, and diminished respiratory muscle performance, which increase the work of breathing and reduce the reserve available when ventilatory demand rises (39). During the 2MST, repeated lower-limb movement increases metabolic and ventilatory demands. Individuals with lower lung-volume-related reserve or weaker respiratory muscles may experience greater relative ventilatory load, breathlessness, or fatigue and may therefore be less able to sustain stepping performance. Respiratory muscle strength has also been prospectively associated with subsequent mobility decline in older adults, supporting a broader connection between respiratory capacity and functional mobility (40). Although the TUG and sit-to-stand tests are shorter in duration, they require rapid integration of lower-limb force production, balance, neuromuscular control, and cardiorespiratory support. Reduced respiratory reserve could therefore plausibly contribute to less efficient task performance. These mechanisms may help explain the observed associations of FVC with the 2MST, TUG, and 30-s chair stand, but they remain hypothetical because ventilation, dyspnea, and respiratory muscle function were not directly assessed.

Forced vital capacity may function as an integrated marker of respiratory and systemic physiological reserve in older adults. FVC is influenced by lung and chest-wall mechanics, respiratory muscle function, body composition, nutritional status, physical activity, comorbidity burden, and the ability to perform a maximal respiratory maneuver (1, 20, 37). It may therefore capture aspects of both pulmonary capacity and general health that are also relevant to physical performance. In contrast, FEV₁/FVC primarily characterizes expiratory airflow limitation relative to the available vital capacity and is particularly useful for identifying obstructive spirometric abnormalities (12). Most participants in the present study were never-smokers, and relatively few had an obstructive spirometric pattern, whereas FVC below the LLN with a preserved FEV₁/FVC ratio was considerably more common. This sample distribution may partly explain why FEV₁/FVC showed weaker associations with physical performance than FVC. However, because total lung capacity was not measured, a low FVC with a preserved FEV₁/FVC ratio cannot confirm restrictive ventilatory impairment. The findings should therefore be interpreted as relating to a non-obstructive low-FVC pattern or reduced lung-volume-related spirometric indices rather than diagnosed restriction (12).

### 4.4 Explanations for low FVC with relatively preserved physical function

In the present study, FVC *z*-scores were generally low, and relatively high proportions of participants had FVC below the LLN or a non-obstructive low-FVC pattern, despite generally preserved baseline mobility. This apparent discrepancy may partly reflect the selected sample and measurement context. Participants were recruited from a health-screening and physical fitness assessment program in a single urban community rather than through population-based sampling; therefore, the proportion below the LLN should not be interpreted as a prevalence estimate for community-dwelling older adults in China. In addition, spirometric measurements depend on maximal inspiration, sustained expiratory effort, participant understanding, and test quality (20). GLI-2012 *z*-scores also depend on the selected reference population (14). Studies in Chinese adults have shown that the choice of reference equation can influence predicted spirometric values and the proportion classified below the LLN (41, 42). Thus, the low FVC distribution observed in this study may reflect a combination of true physiological variation, sample characteristics, test performance, and reference-equation fit. The sensitivity analysis confirmed that the high absolute frequency of low FVC was strongly dependent on the selected reference equation; however, the associations of continuous FVC *z*-scores with TUG and 2MST performance remained directionally consistent when the South East Asian equations were applied. The marked change in LLN-based frequencies, contrasted with the directional consistency of the continuous associations, indicates that equation choice had a greater effect on threshold-based classification than on the overall exposure–outcome pattern. Because the ethnic-group equations use different age-, sex-, and height-specific prediction and dispersion parameters, the resulting *z*-scores are not necessarily related by a single constant linear transformation across participants; modest differences in regression estimates can therefore occur.

Relatively preserved physical performance does not exclude subtle differences in functional reserve. Continuous FVC *z*-scores were associated with TUG, 2MST, and 30-s chair stand performance even though marked mobility limitation was uncommon. In contrast, no adjusted association was observed with the SPPB. Because 82.0% of participants achieved the maximum SPPB score, this null finding may partly reflect a ceiling effect and restricted score variability. Similar limitations of the SPPB have been reported in high-functioning community-dwelling older adults (43, 44).

### 4.5 Clinical and public health implications

Spirometric *z*-scores and objective physical performance tests provide distinct but complementary information. FVC *z*-scores reflect lung-volume-related reserve, whereas the TUG, 2MST, and chair stand tests directly assess mobility, endurance-related performance, and functional lower-limb strength. In this cross-sectional context, their combined use may help characterize concurrent respiratory and functional status, but it cannot identify early decline, establish temporal prediction, or demonstrate a causal effect of pulmonary function on subsequent functional impairment. Previous longitudinal evidence, rather than the present findings, suggests that reduced spirometric function may predict subsequent functional decline and other adverse outcomes in very old adults (45).

However, FVC *z*-scores should not be used alone to diagnose physical functional impairment. They are more appropriately considered complementary markers alongside direct performance testing and clinical assessment. Future longitudinal studies should examine whether FVC *z*-scores predict functional decline, while intervention studies should assess whether respiratory muscle training and broader exercise programs produce parallel improvements in pulmonary and physical outcomes. Inspiratory muscle training can improve respiratory muscle strength, although its effects on functional capacity remain uncertain (46), and structured physical activity may improve mobility without corresponding improvements in FEV₁ or respiratory muscle strength (47). If future prospective and intervention studies establish benefit, exercise and rehabilitation programs could combine individualized goal setting, gradual progression, regular feedback, and follow-up to support long-term adherence. Patient preferences, engagement, acceptability, digital literacy, and access should be considered when selecting center-based, home-based, or hybrid delivery. Evidence from cardiac rehabilitation suggests that immersive virtual-reality approaches may enhance motivation and the rehabilitation experience, while digitally supported or telerehabilitation programs require usable technology, structured clinical support, and attention to social and implementation barriers (48–50). However, these approaches have not been evaluated by the present study, and their transferability to community-dwelling older adults with low spirometric indices requires direct investigation.

### 4.6 Strengths and limitations

This study used GLI-2012 standardized *z*-scores to improve comparability across individuals and assessed physical performance across several domains, including mobility, endurance-related performance, overall lower-extremity function, and functional strength. The use of continuous *z*-scores and LLN classifications also allowed the findings to be examined from complementary analytical perspectives. Standardized testing procedures and adjustment for major demographic and health-related factors further strengthened the analysis.

Several limitations should be acknowledged. First, the cross-sectional design does not allow causal or temporal relationships to be established. Second, participants were recruited from a single urban community through convenience sampling, and most were women, which may limit generalizability and subgroup analyses. Third, spirometry was performed with a portable device in a community setting. FVC is obtained from a maximal forced expiratory maneuver and is therefore effort dependent; submaximal inspiration or expiration can reduce the measured value (20). The field-based physical performance measures likewise require active participant engagement; for example, walking-test performance can vary with verbal encouragement and test administration (51). Because both sets of measurements depend partly on participant effort, shared variation in motivation or engagement could have influenced the observed associations. Although quality-control procedures and standardized test instructions were followed, such effort-related variation cannot be fully excluded. Total lung capacity and respiratory muscle strength were not assessed, so low FVC could not be clearly attributed to restrictive impairment, reduced lung volume, or respiratory muscle weakness. In addition, the ceiling effect of the SPPB may have reduced its ability to detect subtle functional differences. Residual confounding from physical activity, nutritional status, muscle mass, inflammation, and socioeconomic factors may also remain. Finally, the chair-stand analyses were exploratory, and the absence of formal correction for multiple comparisons increases the possibility of chance findings. Larger multicenter longitudinal studies are needed to confirm these results. Residual confounding remains possible despite the physical-activity sensitivity analysis because activity was self-reported and other behavioral or socioeconomic factors were not comprehensively measured. The convenience sample was predominantly female, included few current or former smokers, and was recruited during community health and fitness assessments; healthier and more health-conscious individuals may therefore have been overrepresented, limiting generalizability to the wider older population and to men, smokers, and individuals with poorer health.

## 5 Conclusion

Among community-dwelling older adults, spirometric *z*-scores were associated with multiple domains of physical performance, with the FVC *z*-score showing the most consistent associations. Higher FEV₁ and FVC *z*-scores were associated with shorter TUG times, while a higher FVC *z*-score was also associated with better 2MST and 30-s chair stand performance. Consistently, FVC below the LLN was associated with longer TUG times and poorer 2MST and 30-s chair stand performance, whereas FEV₁/FVC below the LLN was not associated with the core outcomes.

These findings suggest that FVC may provide complementary information on mobility, endurance-related performance, and functional lower-limb strength. Continuous *z*-scores reflect gradations across the pulmonary function distribution, whereas LLN classification identifies values outside the reference range. From a public health and healthy-aging perspective, integrating standardized spirometry with brief physical-performance measures in community health assessments may help flag older adults with concurrent respiratory and functional vulnerability for closer monitoring and preventive support, although prospective validation is required before FVC can be used for risk prediction. If confirmed prospectively, these findings could inform the targeting and evaluation of community-based non-pharmacological approaches—including respiratory muscle training, multicomponent exercise, or combined programs—aimed at preserving mobility, functional independence, and quality of life. Given the single-community cross-sectional design, these findings should be confirmed in larger multicenter longitudinal studies with more balanced sex distributions.

## Data Availability

The raw data supporting the conclusions of this article will be made available by the authors, without undue reservation.
