# Supplementary material for: Associations of spirometric z-scores with mobility, endurance-related performance, and functional lower-limb strength in community-dwelling older adults: a cross-sectional study
Source: Front Public Health. 2026 Sep 23;14:1913269. doi: 10.3389/fpubh.2026.1913269 (PMC13641998; doi:10.3389/fpubh.2026.1913269)
Supplement: Supplementary file 1 [file Data_sheet_1.docx]

Supplementary Material

# 1 Supplementary Data

# 2 Supplementary Figures and Tables

## **Supplementary Figures**

**Supplementary Figure 1. Distributions of spirometric *z*-scores in the study population.**

Kernel density plots show the distributions of FEV₁, FVC, and FEV₁/FVC *z*-scores among 141 community-dwelling older adults. The dashed vertical line indicates the lower limit of normal (LLN), defined as *z*=−1.645. Mean±*SD* values and the number and proportion of participants below the LLN are shown for each spirometric index. FEV₁, forced expiratory volume in 1 second; FVC, forced vital capacity; LLN, lower limit of normal; *SD*, standard deviation.

## **Supplementary Tables**

**Supplementary Table 1. Multivariable linear regression models for SPPB**

| **Outcome** | **Exposure** | ***β* (95% *CI*)** | ***Raw P value*** | ***n*** | **Adjusted R²** |
| --- | --- | --- | --- | --- | --- |
| SPPB | FEV₁ *z*-score | -0.015 (-0.100, 0.069) | 0.719 | 137 | 0.143 |
| SPPB | FVC *z*-score | 0.019 (-0.069, 0.107) | 0.668 | 137 | 0.143 |
| SPPB | FEV₁/FVC *z*-score | -0.028 (-0.086, 0.030) | 0.341 | 137 | 0.148 |

Note. Models were adjusted for age, sex, BMI, smoking status, and comorbidity burden. Each spirometric *z*-score was entered into a separate model. SPPB, Short Physical Performance Battery; *CI*, confidence interval. Raw P values are shown; this secondary SPPB analysis was not included in the prespecified FDR correction.

**Supplementary Table 2. Exploratory regression models for functional lower-limb strength outcomes**

| **Outcome** | **Exposure** | **Adjusted β (95% CI)** | ***Raw P value*** | ***n*** |
| --- | --- | --- | --- | --- |
| 30-s chair stand, repetitions | FEV₁ *z*-score | 0.641 (0.064, 1.218) | 0.030 | 138 |
|  | FVC *z*-score | 1.143 (0.568, 1.718) | <0.001 | 138 |
|  | FEV₁/FVC *z*-score | -0.346 (-0.750, 0.059) | 0.093 | 138 |
| Five-times sit-to-stand, s | FEV₁ *z*-score | -0.188 (-0.582, 0.206) | 0.347 | 138 |
|  | FVC *z*-score | -0.350 (-0.755, 0.055) | 0.090 | 138 |
|  | FEV₁/FVC *z*-score | 0.104 (-0.171, 0.378) | 0.456 | 138 |

Values are unstandardized regression coefficients *β* (95% confidence intervals). The adjusted model included age, sex, body mass index, smoking status, and comorbidity burden. Each spirometric *z*-score was entered into a separate model. FEV₁, forced expiratory volume in 1 second; FVC, forced vital capacity; *CI*, confidence interval. Raw P values are shown; these secondary or exploratory analyses were not included in the prespecified FDR correction.

**Supplementary Table 3. Associations of LLN-defined low spirometric indices with physical performance outcomes**

| **Outcome** | **LLN-defined exposure** | **Adjusted β (95% CI)** | ***Raw P value*** | ***n*** |
| --- | --- | --- | --- | --- |
| Timed Up and Go, s | FEV₁ below LLN | 0.322 (-0.071, 0.714) | 0.108 | 138 |
|  | FVC below LLN | 0.461 (0.061, 0.861) | 0.024 | 138 |
|  | FEV₁/FVC below LLN | 0.443 (-0.135, 1.020) | 0.132 | 138 |
| 4-m gait speed, m/s | FEV₁ below LLN | -0.020 (-0.096, 0.057) | 0.609 | 138 |
|  | FVC below LLN | -0.073 (-0.150, 0.005) | 0.065 | 138 |
|  | FEV₁/FVC below LLN | 0.032 (-0.081, 0.144) | 0.578 | 138 |
| 2-minute step test, repetitions | FEV₁ below LLN | -5.758 (-12.341, 0.824) | 0.086 | 138 |
|  | FVC below LLN | -7.966 (-14.671, -1.262) | 0.020 | 138 |
|  | FEV₁/FVC below LLN | -3.391 (-13.154, 6.373) | 0.493 | 138 |
| SPPB score | FEV₁ below LLN | 0.074 (-0.176, 0.324) | 0.561 | 138 |
|  | FVC below LLN | -0.081 (-0.338, 0.176) | 0.535 | 138 |
|  | FEV₁/FVC below LLN | -0.145 (-0.512, 0.222) | 0.437 | 138 |
| 30-s chair stand, repetitions | FEV₁ below LLN | -0.970 (-2.659, 0.720) | 0.258 | 138 |
|  | FVC below LLN | -2.818 (-4.493, -1.143) | 0.001 | 138 |
|  | FEV₁/FVC below LLN | -0.871 (-3.360, 1.618) | 0.490 | 138 |
| Five-times sit-to-stand, s | FEV₁ below LLN | -0.083 (-1.225, 1.060) | 0.887 | 138 |
|  | FVC below LLN | 0.647 (-0.522, 1.816) | 0.275 | 138 |
|  | FEV₁/FVC below LLN | -0.363 (-2.040, 1.314) | 0.669 | 138 |

Values are unstandardized regression coefficients *β* (95% confidence intervals). Each LLN-defined spirometric index was entered into a separate model, with the corresponding index at or above the LLN as the reference category. The adjusted model included age, sex, body mass index, smoking status, and comorbidity burden. LLN was defined as a *z*-score < −1.645. FEV₁, forced expiratory volume in 1 second; FVC, forced vital capacity; LLN, lower limit of normal; SPPB, Short Physical Performance Battery. Raw P values are shown; these secondary or exploratory analyses were not included in the prespecified FDR correction.

**Supplementary Table 4. Sensitivity analyses using the GLI-2012 South East Asian reference equations**

**A. Spirometric classification according to reference equation**

| **Reference equation** | **FVC *z*-score, mean ± *SD*** | **FVC below LLN, *n* (%)** | **Non-obstructive low-FVC pattern, *n* (%)** | **FEV₁/FVC below LLN, *n* (%)** |
| --- | --- | --- | --- | --- |
| GLI-2012 North East Asian | −2.09 ± 1.39 | 91 (64.5) | 78 (55.3) | 17 (12.1) |
| GLI-2012 South East Asian | −1.01 ± 1.11 | 37 (26.2) | 33 (23.4) | 25 (17.7) |

**B. Adjusted associations using the South East Asian equations**

| **Outcome** | **Exposure** | ***β* (95% *CI*)** | ***Raw P value*** | ***n*** |
| --- | --- | --- | --- | --- |
| TUG time, s | FVC *z*-score | −0.201 (−0.372, −0.031) | 0.021 | 137 |
| 4-m gait speed, m/s | FVC *z*-score | 0.032 (0.000, 0.065) | 0.053 | 137 |
| 2MST, repetitions | FVC *z*-score | 4.274 (1.442, 7.106) | 0.003 | 137 |
| TUG time, s | FVC below LLN | 0.379 (−0.059, 0.816) | 0.089 | 137 |
| 4-m gait speed, m/s | FVC below LLN | −0.066 (−0.149, 0.018) | 0.123 | 137 |
| 2MST, repetitions | FVC below LLN | −9.638 (−16.884, −2.391) | 0.010 | 137 |

Values are unstandardized regression coefficients *β* (95% confidence intervals). Models were adjusted for age, sex, BMI, smoking status, and comorbidity burden. LLN was defined as a *z*-score < −1.645. FVC, forced vital capacity; FEV₁, forced expiratory volume in 1 second; LLN, lower limit of normal; TUG, Timed Up and Go; 2MST, 2-minute step test; *CI*, confidence interval. Raw P values are shown; these secondary or exploratory analyses were not included in the prespecified FDR correction.

**Supplementary Table 5. Outcome-specific false-discovery-rate correction for the primary associations**

| **Outcome** | **Exposure** | ***β* (95% *CI*)** | **Raw *P* value** | **FDR-adjusted *q* value** | ***n*** |
| --- | --- | --- | --- | --- | --- |
| TUG time, s | FEV₁ *z*-score | −0.162 (−0.297, −0.028) | 0.019 | 0.029 | 137 |
| TUG time, s | FVC *z*-score | −0.167 (−0.306, −0.028) | 0.019 | 0.029 | 137 |
| TUG time, s | FEV₁/FVC *z*-score | −0.027 (−0.123, 0.069) | 0.575 | 0.575 | 137 |
| 4-m gait speed, m/s | FEV₁ *z*-score | 0.015 (−0.011, 0.041) | 0.247 | 0.371 | 137 |
| 4-m gait speed, m/s | FVC *z*-score | 0.025 (−0.001, 0.052) | 0.064 | 0.192 | 137 |
| 4-m gait speed, m/s | FEV₁/FVC *z*-score | −0.004 (−0.023, 0.014) | 0.652 | 0.652 | 137 |
| 2MST, repetitions | FEV₁ *z*-score | 2.277 (−0.004, 4.558) | 0.050 | 0.075 | 137 |
| 2MST, repetitions | FVC *z*-score | 3.605 (1.297, 5.913) | 0.002 | 0.006 | 137 |
| 2MST, repetitions | FEV₁/FVC *z*-score | −0.440 (−2.058, 1.178) | 0.591 | 0.591 | 137 |

Values are unstandardized regression coefficients *β* (95% confidence intervals) from the adjusted model, including age, sex, BMI, smoking status, and comorbidity burden. The Benjamini–Hochberg procedure was applied separately within each primary-outcome family comprising the three spirometric *z*-score associations. FDR, false discovery rate; TUG, Timed Up and Go; 2MST, 2-minute step test; FEV₁, forced expiratory volume in 1 second; FVC, forced vital capacity; *CI*, confidence interval.

**Supplementary Table 6. Physical-activity-adjusted sensitivity analysis of the primary associations**

| **Outcome** | **Exposure** | ***β* (95% *CI*)** | **Raw *P* value** | **FDR-adjusted *q* value** | ***n*** |
| --- | --- | --- | --- | --- | --- |
| TUG time, s | FEV₁ *z*-score | −0.145 (−0.277, −0.013) | 0.031 | 0.051 | 137 |
| TUG time, s | FVC *z*-score | −0.148 (−0.285, −0.011) | 0.034 | 0.051 | 137 |
| TUG time, s | FEV₁/FVC *z*-score | −0.012 (−0.106, 0.082) | 0.804 | 0.804 | 137 |
| 4-m gait speed, m/s | FEV₁ *z*-score | 0.015 (−0.012, 0.041) | 0.267 | 0.401 | 137 |
| 4-m gait speed, m/s | FVC *z*-score | 0.025 (−0.002, 0.052) | 0.069 | 0.206 | 137 |
| 4-m gait speed, m/s | FEV₁/FVC *z*-score | −0.006 (−0.024, 0.013) | 0.558 | 0.558 | 137 |
| 2MST, repetitions | FEV₁ *z*-score | 1.801 (−0.361, 3.964) | 0.102 | 0.153 | 137 |
| 2MST, repetitions | FVC *z*-score | 3.065 (0.863, 5.267) | 0.007 | 0.020 | 137 |
| 2MST, repetitions | FEV₁/FVC *z*-score | −0.753 (−2.276, 0.771) | 0.330 | 0.330 | 137 |

Values are unstandardized regression coefficients *β* (95% confidence intervals) from models adjusted for age, sex, BMI, smoking status, comorbidity burden, and self-reported physical activity (no regular exercise, occasional exercise, or regular exercise). The Benjamini–Hochberg procedure was applied separately within each primary-outcome family. FDR, false discovery rate; TUG, Timed Up and Go; 2MST, 2-minute step test; FEV₁, forced expiratory volume in 1 second; FVC, forced vital capacity; *CI*, confidence interval.
